# The Interplay of Interstitial and Substitutional Copper in Zinc Oxide

**DOI:** 10.3389/fchem.2021.780935

**Published:** 2021-12-14

**Authors:** Qing Hou, John Buckeridge, Aron Walsh, Zijuan Xie, You Lu, Thomas W. Keal, Jingcheng Guan, Scott M. Woodley, C. Richard A. Catlow, Alexey A. Sokol

**Affiliations:** ^1^ Institute of Photonic Chips, University of Shanghai for Science and Technology, Shanghai, China; ^2^ School of Materials and Chemistry, University of Shanghai for Science and Technology, Shanghai, China; ^3^ Department of Chemistry, Kathleen Lonsdale Materials Chemistry, University College London, London, United Kingdom; ^4^ School of Engineering, London South Bank University, London, United Kingdom; ^5^ Department of Materials, Imperial College London, London, United Kingdom; ^6^ Shenzhen Institute for Quantum Science and Technology and Department of Physics, Southern University of Science and Technology, Shenzhen, China; ^7^ Scientific Computing Department, UKRI STFC Daresbury Laboratory, Warrington, United Kingdom; ^8^ School of Chemistry, Cardiff University, Cardiff, United Kingdom

**Keywords:** zinc oxide, copper, hybrid QM/MM, dopant, defects

## Abstract

Cu impurities are reported to have significant effects on the electrical and optical properties of bulk ZnO. In this work, we study the defect properties of Cu in ZnO using hybrid quantum mechanical/molecular mechanical (QM/MM)–embedded cluster calculations based on a multi-region approach that allows us to model defects at the true dilute limit, with polarization effects described in an accurate and consistent manner. We compute the electronic structure, energetics, and geometries of Cu impurities, including substitutional and interstitial configurations, and analyze their effects on the electronic structure. Under ambient conditions, Cu_Zn_ is the dominant defect in the d^9^ state and remains electronically passive. We find that, however, as we approach typical vacuum conditions, the interstitial Cu defect becomes significant and can act as an electron trap.

## Introduction

ZnO, with a wide bandgap of 3.44 eV (Reynolds et al., 1999), is one of the most widely studied transparent semiconductors, with applications in solar cells (Keis et al., 2002), light-emitting diodes (Tsukazaki et al., 2005a; Tsukazaki et al., 2005b), photocatalysts (Barick et al., 2010), and piezoelectric devices (Wang and Song, 2006). To optimize its performance in applications, it is important to understand and control the properties of impurities in ZnO. The Cu/ZnO system is a very important industrial methanol catalyst (Waugh, 1992; Baltes et al., 2008). Substitution of copper into ZnO (Cu_Zn_^*q*^) (here and elsewhere in this article, we denote the effective charge of the defect, *q*, with respect to the lattice site explicitly with the superscript) has been reported to improve the photocatalytic activity (Mohan et al., 2012), ferromagnetism (Xing et al., 2008), and gas sensitivity (Gong et al., 2006) of ZnO. By admittance spectroscopy experiments, Cu_Zn_^*q*^ is found to possess an (0/−) acceptor level at 0.17 eV below the bottom of the conduction band (CBM) in ZnO (McCluskey et al., 2015). Doping with copper has been proposed as a route for producing stable p-type ZnO (Özgür et al., 2005), which, however, has not been successful to date and which partly inspired our current investigation. Moreover, although there are many investigations both experimental and computational on the effects of CuZn on the electrical and optical properties of ZnO, information about Cu interstitials (Cu_i_^*q*^) in ZnO is limited.

In this article, we report the properties of Cu in both substitutional and interstitial forms in ZnO using a hybrid quantum mechanical/molecular mechanical (QM/MM)–embedded cluster approach. For the description of point defects in crystals, the commonly used implementation of density functional theory (DFT) with periodic boundary conditions suffers from finite-size effects (Freysoldt et al., 2014). In contrast, the QM/MM method defines the vacuum reference level unambiguously and describes accurately the short- and long-range polarization effects of a charged defect in a host material. The approach allows us to compare the energetics of different defect configurations and charge states on an equal footing. We focus here on isolated defects, but copper impurities may also form complexes in a variety of ways, an investigation into which is underway and will be reported elsewhere.

## Computational Techniques

In this work, the QM region containing 86 atoms is treated with DFT using a triple zeta plus polarization Gaussian basis set for oxygens (Def2-TZVPP) (Weigend and Ahlrichs, 2005) and a double zeta plus polarization set for Zn cations (cc-pVDZ-PP) (Peterson, 2003; Figgen et al., 2005) (relevant input files can be found in the git repository “https://github.com/qhou1/chemshell.git”). To reduce the computational load, we have removed *f* functions from the oxygen basis set and some of the highly diffuse functions from the cation basis sets, which do not contribute to the bonding in these ionic solids.

For electron exchange and correlation, we have employed the BB1k functional (Zhao et al., 2004), which has been fitted to both thermochemical and kinetic data including 42% exact exchange, and the PBE0 functional (Adamo and Barone, 1999), which is frequently used in plane-wave basis calculations including 25% exact exchange for comparison. In order to embed the QM cluster within a polar environment, the MM region containing 10,460 atoms is treated with a previously derived interatomic potential (Catlow et al., 2008).

The hybrid QM/MM-embedded cluster approach used is implemented in the ChemShell (Sherwood et al., 2003) package. The QM/MM energy is obtained in an additive approach as a sum of QM and MM terms with the interaction energy between the two regions accounted for the QM term whose Hamiltonian includes the embedding potential. The GAMESS-UK (Guest et al., 2005) code is employed in the QM calculations, while the GULP package has been used to calculate the MM contributions.

The formation energies of the Cu substitution with charge *q* are calculated according to the following reactions:

$$Cu_{\left(s\right)}+Zn_{\mathrm{Zn}}^{0}\to Cu_{\mathrm{Zn}}^{q-}+Zn_{\left(s\right)}+qh^{+},$$ (1)

under Zn-rich/O-poor condition and

$$\mathrm{Cu}O_{\left(s\right)}+Zn_{\mathrm{Zn}}^{0}\to Cu_{\mathrm{Zn}}^{q-}+\mathrm{Zn}O_{\left(s\right)}+qh^{+},$$ (2)

under Zn-poor/O-rich condition.

The formation energies of the Cu interstitial are calculated according to the following reaction:

$$Cu_{\left(s\right)}\to Cu_{i}^{q+}+qe^{-},$$ (3)

under Zn-rich/O-poor condition and

$$\mathrm{Cu}O_{\left(s\right)}\to Cu_{i}^{q+}+\frac{1}{2}O_{2\left(g\right)}+qe^{-},$$ (4)

Under Zn-poor/O-rich condition.

The chemical potentials of O_2_ molecular and single Zn atoms are calculated using GAMESS-UK with the corresponding basis set and density functional; the standard state energy of ZnO is derived from the experimental heat of formation (Haynes, 2014).

## Results and Discussion

We first present the local geometries as well as the defect formation of the Cu interstitial Cu_i_^*q*^ and Cu substitutional Cu_Zn_^*q*^; we next calculate the self-consistent Fermi energies and charge carrier and defect concentrations of Cu-doped ZnO. Finally, we report on the equilibrium between Cu_Zn_^*q*^ and Cu_i_^*q*^.

### Positions of Cu

As noted, Cu impurities have been widely studied due to their possible influence on the optical properties of ZnO. Moreover, by using the emission channeling technique, 60–70% of the Cu atoms are found to occupy the substitutional Zn site with root-mean-square displacements from the site of 0.16–0.17 Å (Wahl et al., 2004).

**FIGURE 1:**
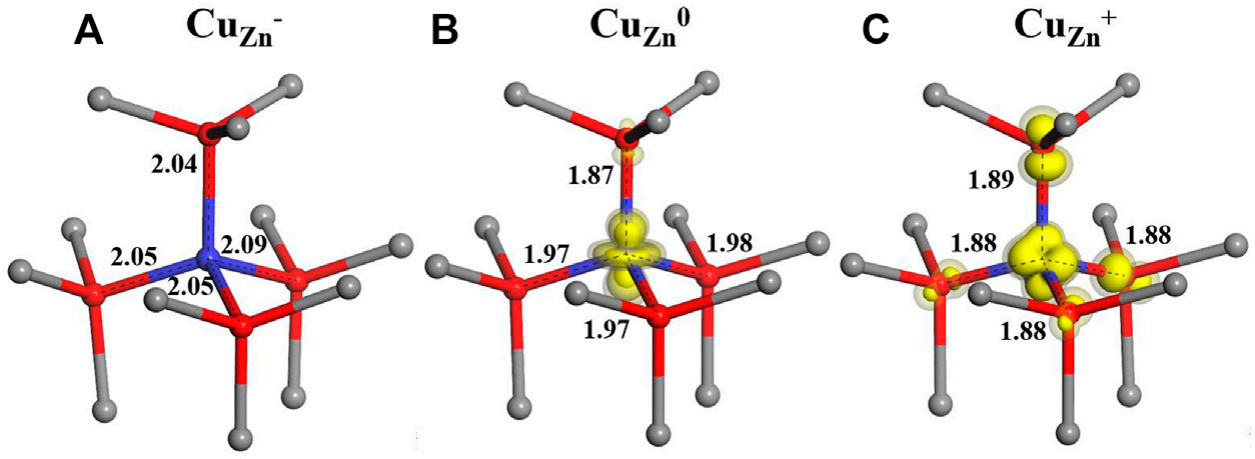
Calculated local structures for three charge states ( **(A)**-1, **(B)**0 and **(C)** +1) of the Cu_Zn_ defect in ZnO, with zinc ions represented by grey spheres, oxygen ions by red spheres and copper ions by blue spheres.

In our calculation, for the singly negatively charged state of Cu_Zn_^−^, the four nearest O neighbors of Cu are displaced outward by 0.11–0.14 Å (using the BB1k functional) as shown in Figure 1A. After an electron is removed from this negatively charged system, the neutrally charged state Cu_Zn_ is formed. As shown by the spin density in Figure 1B, the resulting d^9^ Cu impurity drives a Jahn–Teller distortion, with the axial neighbor O relaxing inward by 0.06 Å and the other three non-axial O ions relaxing outward by 0.03 Å. In the +1 charge state, four neighbor O ions all relax inward by 0.04–0.07 Å around the d^10^ Cu ion (Figure 1C).

The Cu impurities could also be present as interstitials in ZnO, in two possible positions: octahedral and tetrahedral sites. As discussed in previous studies by Janotti and Van de Walle (Janotti and Van de Walle, 2007) and Sokol *et al.* (Sokol et al., 2007), the Zn interstitial is expected to be more stable at the octahedral site than at the tetrahedral site. Hence, here, we only consider the interstitial at the octahedral site.

**FIGURE 2:**
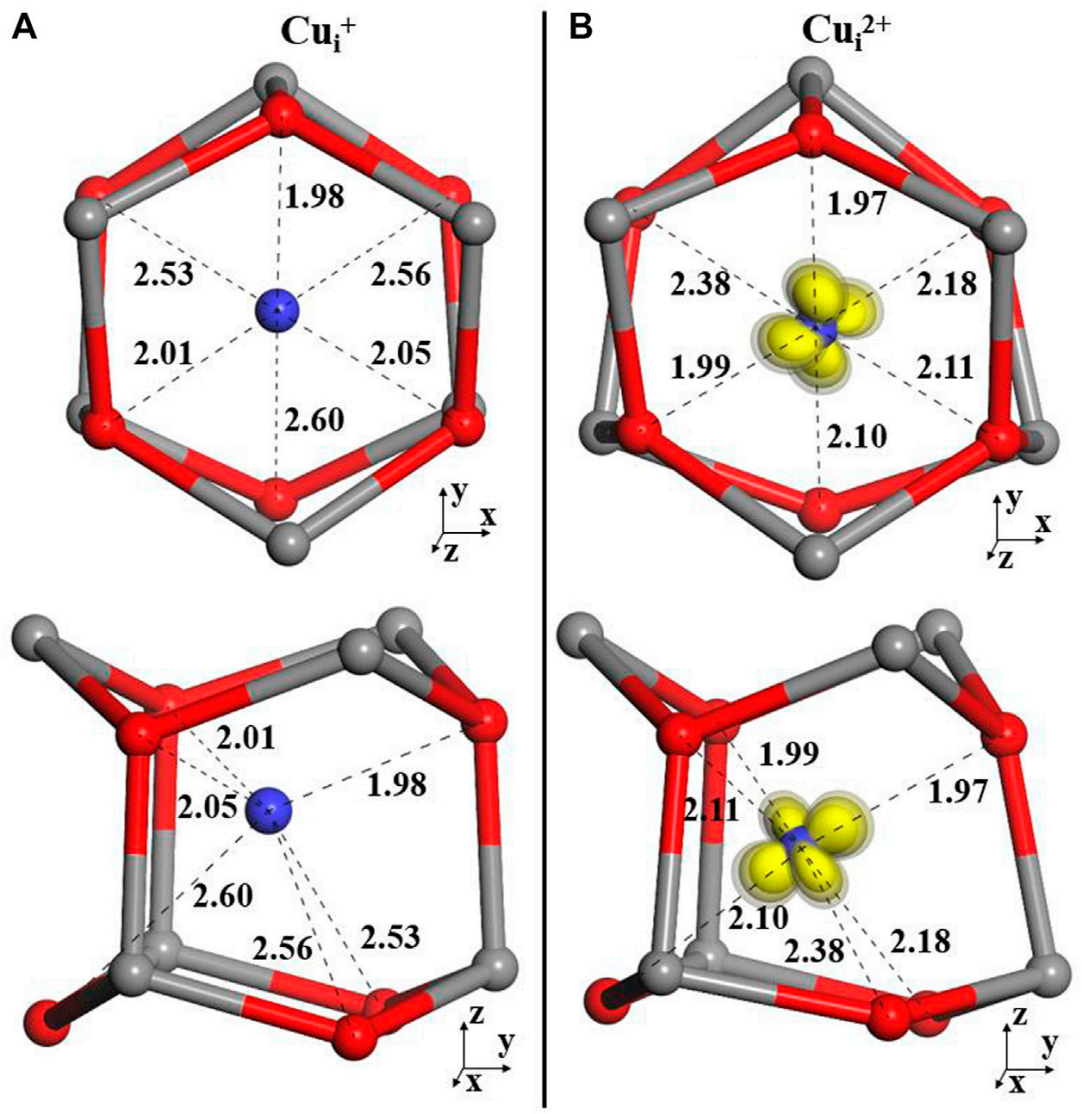
Local structures of Cu_i_ in +1 **(A)** and 2+**(B)** charge states.

The calculated configurations are illustrated in Figure 2B. The Cu^+^ interstitial has a lower coordination by electron-rich O^2-^ ions and forms a trigonal pyramid with the closest Cu_i_–O separation distance of 1.98 Å and the two other distances of 2.01 and 2.05 Å (BB1k structures are shown in Figure 2A). On ionization, this nearly symmetric configuration is broken, with the Cu^2+^ ion moving toward one of the lattice oxygens (1.97, 1.98, and 2.11 Å). The next nearest O ions move now toward the interstitial Cu (by 0.16, 0.50, and 0.38 Å) but do not approach close enough to coordinate to this ion directly by a dative bond.

### Formation Energies

**FIGURE 3:**
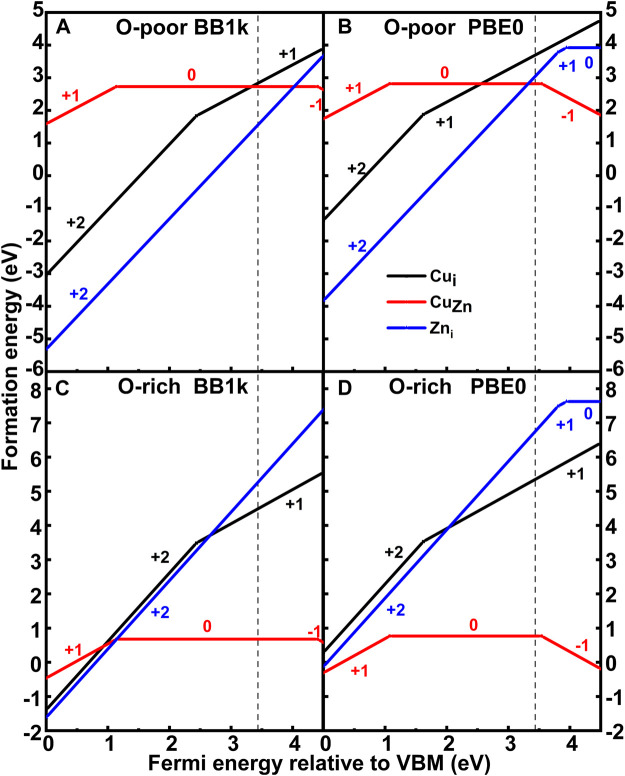
Formation energy of Cu_Zn_ and Cu_i_ in ZnO as a function of the Fermi level relative to the valence band maximum (VBM) under O-poor (**(A)** BB1k and **(B)** PBE0) and O-rich (**(C)** BB1k and **(D)** PBE0) conditions.

The calculated formation energies of both Cu_Zn_ and Cu_i_ are plotted in Figure 3. The (0/−) transition level of Cu_Zn_^*q*^ is found to lie at 3.54 eV (PBE0) above the valence band maximum (VBM), which is in agreement with previous calculations by Lany and Zunger (Lany and Zunger, 2009), who reported 3.46 eV using generalized gradient approximation (GGA)+*U* with an additional hole-state correction for the Cu *d* state, and close to the calculations by Lyons *et al.* (Lyons et al., 2017), who reported 3.27 eV using the Heyd–Scuseria–Ernzerhof (HSE) hybrid functional. Our results contrast with Yan et al. (Yan et al., 2006), who reported 0.7 eV using local density approximation (LDA), and Gallino and Valentin (Gallino and Di Valentin, 2011), who reported 2.48 eV using B3LYP. The computed ε(0/−) using BB1k is, however, 4.40 eV. The (+/0) transition level of Cu_Zn_^*q*^ is found to lie at 1.14 eV (BB1k) and 1.07 eV (PBE0) above the VBM, which yields a deep donor level, which is shallower than that of Lany and Zunger (Lany and Zunger, 2009), who reported 0.37 eV, and Lyons et al. (Lyons et al., 2017), who reported 0.46 eV.

In O-poor conditions, we observe that the Cu_i_^*q*^ is more stable than Cu_Zn_^*q*^ using BB1k until the Fermi level is very close to the CB from the calculated formation energies. In O-rich conditions, when the Fermi level is near the VBM, the interstitial Cu is still more stable than substitutional Cu. While for PBE0 results, the substitutional Cu becomes more stable than the interstitial Cu for the Fermi level greater than 2.56 eV under the Zn-rich condition. Under O-rich conditions, the substitutional Cu is the most stable defect type in the d^9^ state as a donor.

### Charge Carrier and Defect Concentrations

From the computed formation energies, the self-consistent Fermi energy and equilibrium defect and carrier concentrations can be determined. Here, we use the code “SC-FERMI” (Buckeridge, 2019). We focus on the results obtained using the BB1k functional, which reproduces the localization of holes on the oxygen sublattice more accurately than that on other functionals we have tested.

The concentration of each defect X in each charge state q is given by:

$$C_{X^{q}}=N_{X}g_{X^{q}}\exp\left(-\frac{E_{f}\left(X^{q}\right)}{\mathrm{kT}}\right),$$ (7)

where *N*_X_ is the density of sites in which the defect may form, g_X_^*q*^ is the degeneracy of the charge state, *E*_*F*_ is the self-consistent Fermi energy, and *k* is the Boltzmann constant.

The electron (*n*_0_) and hole (*p*_0_) carrier concentrations can be determined by integrating the density of states weighed by the appropriate Fermi–Dirac function:

$$n_{0}=\overset{\infty }{\underset{E_{g}}{\int }}f_{e}\left(E\right)\rho \left(E\right)\mathrm{dE;}$$ (8)

$$p_{0}=\overset{0}{\underset{-\infty }{\int }}f_{h}\left(E\right)\rho \left(E\right)\mathrm{dE,}$$ (9)

where $f_{\text{e}}\left(E\right)$ = [exp ((*E*_*F*_ *-E*)/*k*T) +1]^−1^ is the Fermi–Dirac distribution function and $f_{\text{h}}\left(E\right)$ = 1- $f_{\text{e}}\left(E\right)$ .

**FIGURE 4:**
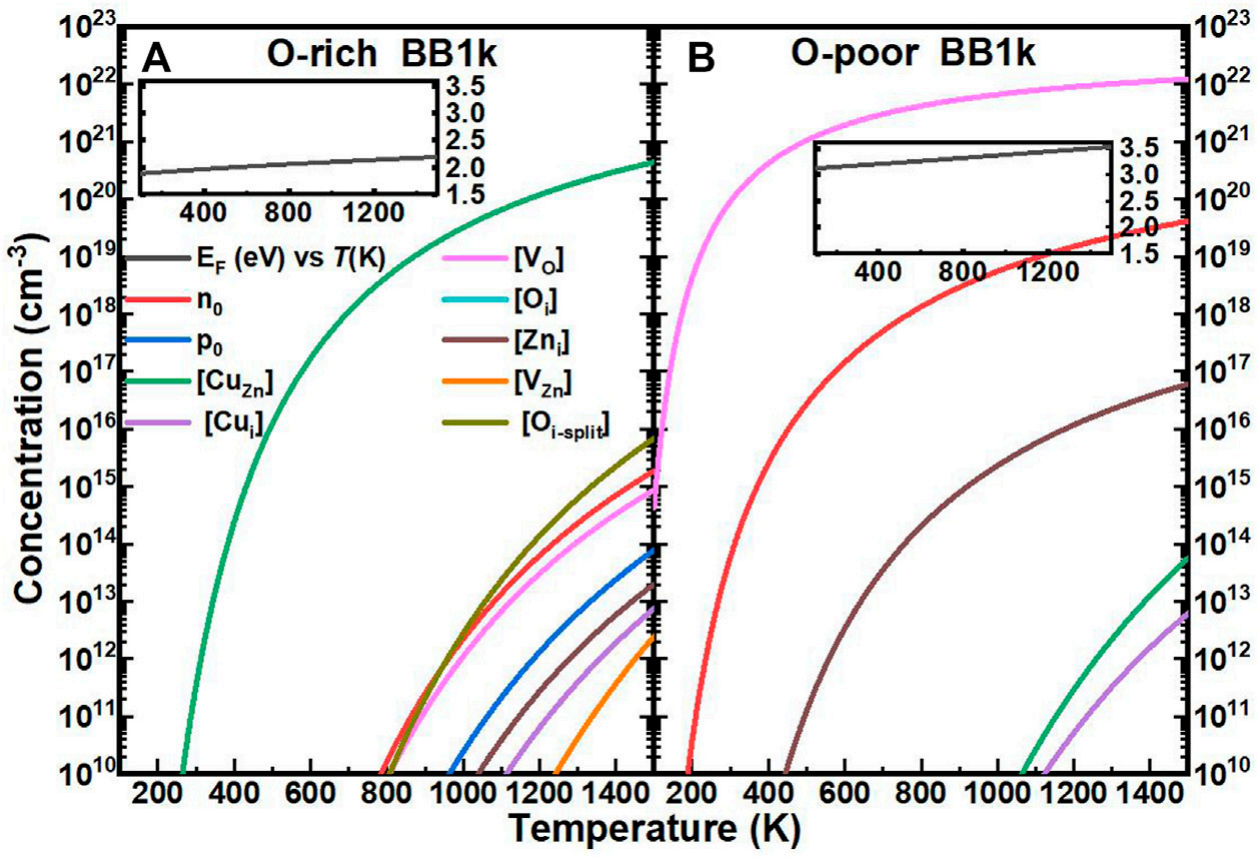
Calculated self-consistent Fermi energy (E_F_, relative to the CBM, black line) and equilibrium concentrations of electrons (n_0_, red line), holes (p_0_, blue line), Cu substitutionals ([Cu_Zn_], green line), Cu interstitials ([Cu_i_], purple line), oxygen vacancies ([V_O_], magenta line), oxygen interstitials ([O_i_], cyan line), zinc interstitials ([Zn_i_], brown line), zinc vacancies ([V_Zn_], orange line), and oxygen split interstitials ([O_i_], dark yellow line)in ZnO as a function of temperature, determined using the BB1k hybrid density functional, under O-rich **(A)** and O-poor **(B)** conditions.

The computed self-consistent *E*_*F*_ and equilibrium carrier and equilibrium concentrations of Cu impurities with native defects in ZnO as a function of T are shown in Figure 4. The range of temperatures is 0–1500 K, which encompasses common synthesis temperatures of ZnO and a majority of device operational temperatures.

In O-rich conditions, the *E*_*F*_ remains deep in the bandgap, between 1.9 and 2.2 eV above the VBM as *T* is increased, as shown in the inset of Figure 4A. The carrier concentrations remain below 10^16^ cm^−3^ for *T* ≤ 1500 K, with the Cu interstitial concentration [Cu_i_] three-order of magnitude below. The [Cu_Zn_] in the neutrally charged state is the dominant defect in this range of *E*_*F,*_ with the concentration above 10^18^ cm^−3^ for *T* > 600 K, which is close to the experimental result of ∼10^18^ cm^−3^ at room temperature (Kanai, 1991).

In O-poor conditions, due to the lower formation energies, *E*_*F*_ moves closer to the CB and even above the CBM as shown in the inset of Figure 4B. From our analysis, ZnO is found to be *n*-type with electron concentrations n_0_ of 10^16^ cm^−3^ for T > 453 K (Hou, 2021) (details of the properties of native defects in ZnO will be published in the future).

**FIGURE 5:**
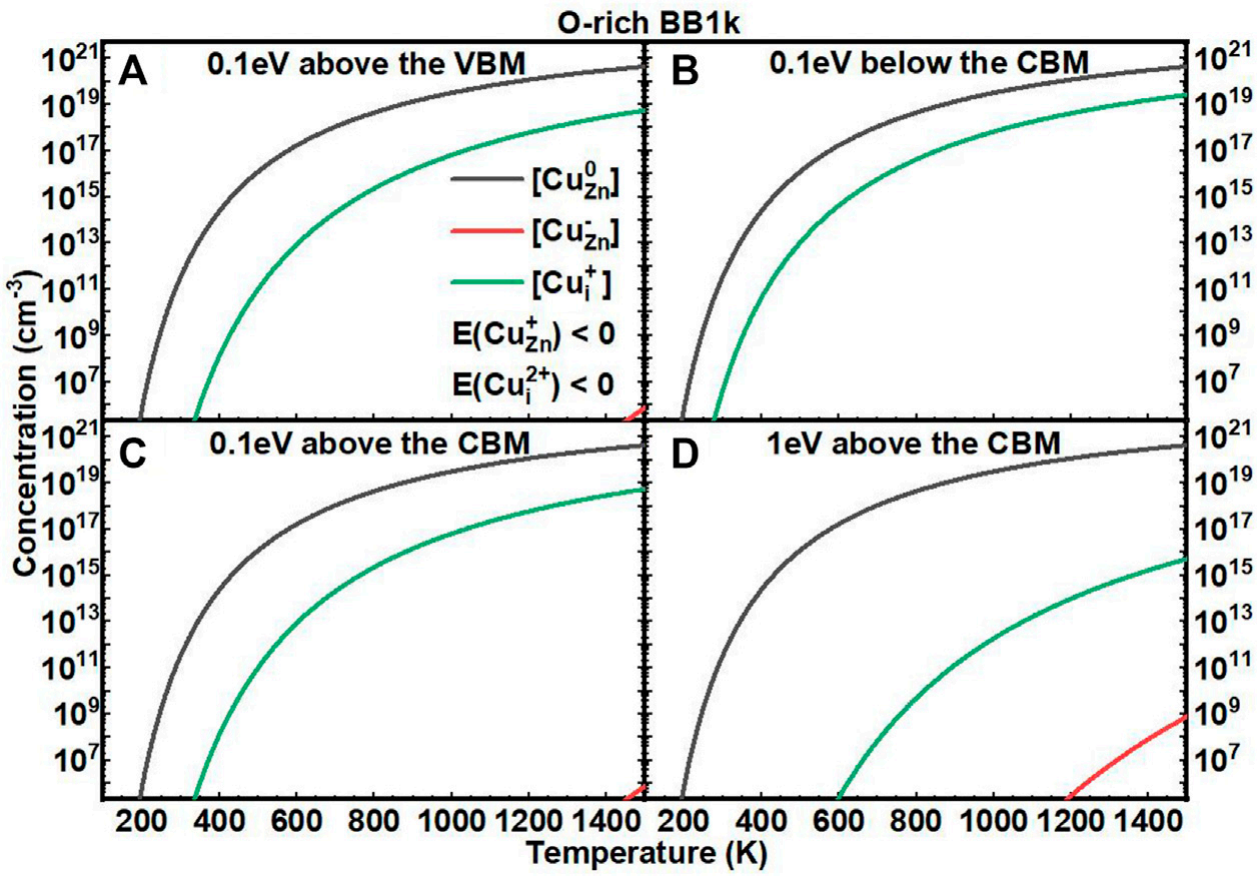
Calculated equilibrium concentrations of Cu substitutional in the 0 charge state ([Cu_Zn_^0^], black line), Cu substitutional in the −1 charge state ([Cu_Zn_^−^], red line), Cu substitutional in the +1 charge state ([Cu_Zn_^+^], blue line), Cu interstitial in the +1 charge state ([Cu_Zn_^+^], green line), and Cu interstitial in the +2 charge state ([Cu_Zn_^2+^], purple line) in ZnO as a function of temperature at a fixed fermi level (**(A)** 0.1eV above the VBM, **(B)** 0.1eV below the CBM, **(C)** 0.1eV above the CBM, **(D)** 1eV above the CBM), determined using the BB1k hybrid density functional, under O-rich conditions.

**FIGURE 6:**
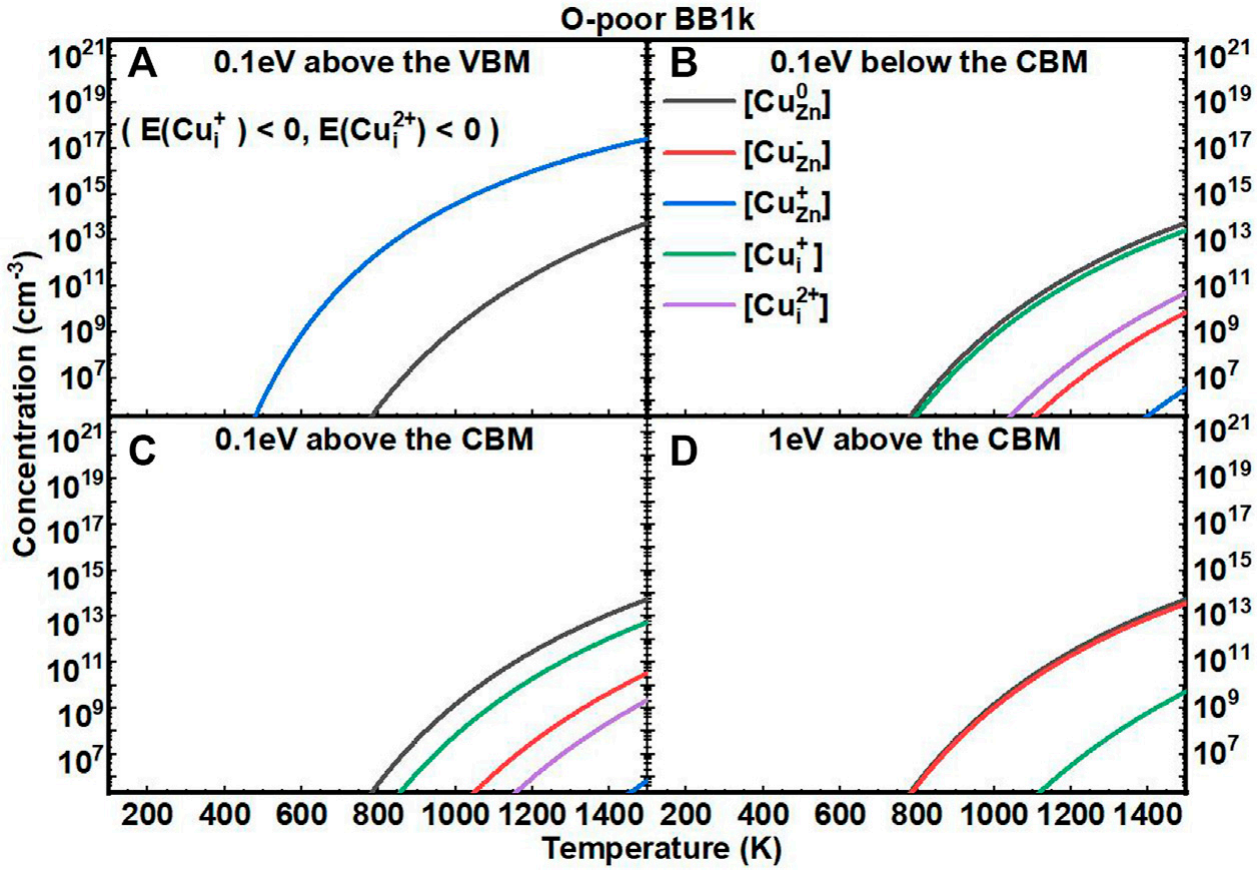
Calculated equilibrium concentrations of Cu substitutional in the 0 charge state ([Cu_Zn_^0^], black line), Cu substitutional in the −1 charge state ([Cu_Zn_^−^], red line), Cu substitutional in the +1 charge state ([Cu_Zn_^+^], blue line), Cu interstitial in the +1 charge state ([Cu_Zn_^+^], green line), and Cu interstitial in the +2 charge state ([Cu_Zn_^2+^], purple line) in ZnO as a function of temperature at a fixed fermi level (**(A)** 0.1eV above the VBM, **(B)** 0.1eV below the CBM, **(C)** 0.1eV above the CBM, **(D)** 1eV above the CBM), determined using the BB1k hybrid density functional, under O-poor conditions.

We next investigated the equilibrium defect concentrations of Cu impurities in ZnO with fixed *E*_*F*_ as a function of T (Figures 5, 6). We mainly considered four conditions of *E*_*F*_ at: (A) 0.1 eV above the VBM, (B) 0.1 eV below the CBM, (C) 0.1 eV above the CBM, and (D) 1 eV above the CBM. We note here that the concentrations are not available when the formation energies of the defects are negative based on Eq. 7. In O-rich conditions, the [Cu_Zn_] in the neutrally charged state is the dominant defect for all four conditions. In O-poor conditions, when the *E*_*F*_ is near to the CBM, the [Cu_i_^+^] is close to [Cu_Zn_^0^], but both are in relatively low concentrations.

### O Partial Pressure Variation

To compare our results to the experiment, it is important to relate the theoretically defined O-rich and O-poor condition to the oxygen chemical potential under different temperature and partial pressure conditions. The chemical potential of oxygen gas at varying oxygen partial pressures at a given temperature is expressed by:

$$\mu_{O}\left(T,p\right)=\mu_{O}\left(T,p^{0}\right)+\frac{1}{2}\mathrm{kT}\ln\frac{p}{p^{0}}.$$ (10)

By setting the zero state of $\mu_{\text{O}}\left(T,p\right)$ to be the total energy of oxygen at *T* = 0 K, which is $\mu_{O}\left(0,p^{0}\right)=1/2E_{O_{2}}^{\mathrm{total}}=0$ , the temperature dependence of the oxygen chemical potential at a constant oxygen pressure *p* ^0^ is defined as:

$$\mu_{O}\left(T,p^{0}\right)=\frac{1}{2}\left[H\left(T,p^{0},O_{2}\right)-H\left(0,p^{0},O_{2}\right)\right]-\frac{1}{2}T\left[S\left(T,p^{0},O_{2}\right)-S\left(0,p^{0},O_{2}\right)\right]\;,$$ (11)

where *H* is the enthalpy, and *S* is the entropy. Based on the data from thermochemical tables (Stull and Prophet, 1971), $\mu_{\text{O}}\left(T,p^{0}\right)$ at $p^{0}=1\;\text{atm}$ was calculated by Taylor *et* al. (Taylor et al., 2016) and Reuter and Scheffler (Reuter and Scheffler, 2001) ( $\mu_{O}\left(300,p^{0}\right)=-0.27\;\mathrm{eV}$ ; $\;\mu_{\text{O}}\left(1000,p^{0}\right)=-1.01\text{ eV}$ ).

**FIGURE 7:**
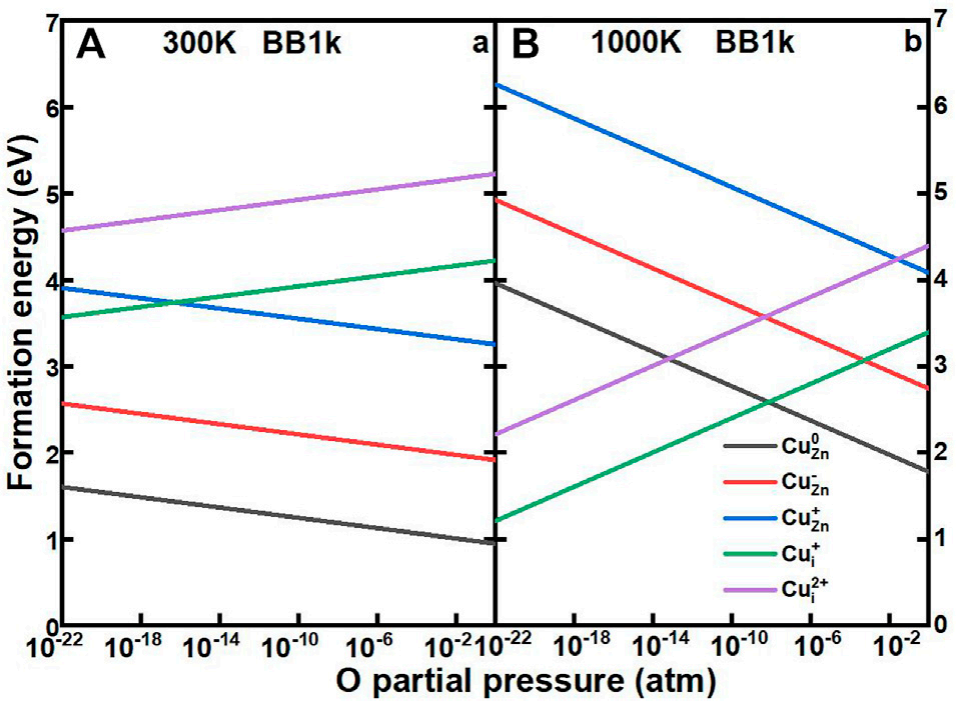
Calculated formation energies of Cu_Zn_^0^ (black line), Cu_Zn_^−^ (red line), Cu_Zn_^+^ (blue line), Cu_Zn_^+^ (green line), and Cu_Zn_^2+^ (purple line) in ZnO as a function of oxygen partial pressures, determined using the BB1k hybrid density functional, under 300K **(A)** and 1000 K **(B)**>.

Our procedure then follows the method by Reuter and Scheffler (Reuter and Scheffler, 2001). The formation energies of Cu impurities in ZnO as a function of the O partial pressure from 10^–22^ to 1 atm at 300 and 1000 K are shown in Figure 7. At 300 K, the Cu_Zn_ in the neutrally charged state remains as the dominant defect for O partial pressures from 10^–22^ to 1 atm, which is consistent with the defect concentration results. At high temperatures of 1000 K, the Cu_i_^+^ becomes the dominant defect under very low O partial pressures, with the crossover from Cu_Zn_^0^ to Cu_i_^+^ at 6.5*10^–9^ atm.

### Balance Between Cu Substitutional and Interstitial

Bulk ZnO contains significant concentrations of intrinsic defects, to which are attributed the intrinsic *n*-type conductivity in ZnO (Harrison, 1954; Thomas, 1957; Hagemark, 1976). In the presence of the Cu dopants, there can be interchange of electrons or holes between Cu substitutional with extrinsic defects and Cu interstitial with intrinsic defect Zn vacancy or interstitial, for *e.g.,*

$$Cu_{\mathrm{Zn}}^{0}+V_{i}^{0}\to Cu_{i}^{q+}+V_{\mathrm{Zn}}^{p-}+\left(q-p\right)e^{-};$$ (12)

$$Cu_{\mathrm{Zn}}^{0}+Zn_{i}^{r+}\to Cu_{i}^{q+}+Zn_{\mathrm{Zn}}^{0}+\left(r-q\right)h^{+}.$$ (13)

The corresponding processes and their reaction energies ΔE_*f*_ (in eV) are listed in Table 1, with the electron in the CB and the hole in the VB. In general, Cu_Zn_^0^ remains energetically preferable. Cu will not migrate directly from the substitutional to the interstitial site under ambient conditions, owing to the high reaction energy at 6.21 and 6.15 eV. However, under O-poor/Zn-rich conditions, in the presence of the native defect Zn interstitial, Cu will spontaneously migrate to the interstitial site with a reaction energy of −0.45 eV; the Cu_i_ can trap electrons from CB.

**TABLE 1:** Reaction energies (eV) for Cu_Zn_ and Cu_i_ with intrinsic defect V_Zn_ or Zn_i_.

Defect reaction	Δ*E*_f_ (eV)	
	$e^{-}$ in the CB	$h^{+}$ in the VB
$Cu_{Zn}^{0}+V_{i}^{0}\to Cu_{i}^{2+}+V_{\mathrm{Zn}}^{2-}$	6.21	6.21
$Cu_{Zn}^{0}+V_{i}^{0}\to Cu_{i}^{2+}+V_{Zn}^{-}+e^{-}$	7.15	n/a
$Cu_{Zn}^{0}+V_{i}^{0}\to Cu_{i}^{2+}+V_{Zn}^{0}+2e^{-}$	8.19	n/a
$Cu_{Zn}^{0}+V_{i}^{0}\to Cu_{i}^{2+}+V_{Zn}^{+}+3e^{-}$	9.78	n/a
$Cu_{Zn}^{0}+V_{i}^{0}\to Cu_{i}^{2+}+V_{Zn}^{2+}+4e^{-}$	12.10	n/a
$Cu_{Zn}^{0}+V_{i}^{0}\to Cu_{i}^{+}+V_{Zn}^{2-}+h^{+}$	n/a	8.65
$Cu_{Zn}^{0}+V_{i}^{0}\to Cu_{i}^{+}+V_{Zn}^{-}$	6.15	6.15
$Cu_{Zn}^{0}+V_{i}^{0}\to Cu_{i}^{+}+V_{Zn}^{0}+e^{-}$	7.19	n/a
$Cu_{Zn}^{0}+V_{i}^{0}\to Cu_{i}^{+}+V_{Zn}^{+}+2e^{-}$	8.78	n/a
$Cu_{\mathrm{Zn}}^{0}+V_{i}^{0}\to Cu_{i}^{+}+V_{\mathrm{Zn}}^{2+}+3e^{-}$	11.10	n/a
$Cu_{\mathrm{Zn}}^{0}+Zn_{i}^{2+}\to Cu_{i}^{+}+Zn_{Zn}^{0}+h^{+}$	n/a	−1.46
$Cu_{\mathrm{Zn}}^{0}+Zn_{i}^{2+}\to Cu_{i}^{2+}+Zn_{Zn}^{0}$	−0.45	−0.45

### Summary and Conclusion

We have investigated the copper dopants in both substitutional and interstitial forms in ZnO from embedded cluster calculations. By computing defect formation energies, we find that the Cu substitutional in the neutrally charged state is the dominant defect under O-rich conditions, which acts as a deep donor, while under Zn-rich conditions Cu interstitial becomes more stable than the Cu substitutional, which is consistent with the results of the equilibrium carrier and effect concentrations as a function of temperature. The Cu will not migrate directly from the substitutional to the interstitial site under ambient conditions, but under Zn-rich conditions Cu will spontaneously migrate to the interstitial site and trap electrons in the presence of Zn interstitial.

## Data Availability

The original contributions presented in the study are included in the article/Supplementary Material; further inquiries can be directed to the corresponding authors.
